# Hydrogen Peroxide Is Involved in *β*-Cyclodextrin-hemin Complex-Induced Lateral Root Formation in Tomato Seedlings

**DOI:** 10.3389/fpls.2017.01445

**Published:** 2017-08-18

**Authors:** Weiti Cui, Dan Zhu, Wenbiao Shen, Yudong Mei, Dekun Hu, Yujian Shi, Yong Ren, Wei Shen, Quan Gu, Daokun Xu, Liqin Huang

**Affiliations:** ^1^Laboratory Center of Life Sciences, College of Life Sciences, Nanjing Agricultural University Nanjing, China; ^2^College of Sciences, Nanjing Agricultural University Nanjing, China; ^3^College of Life Sciences, Nanjing Normal University Nanjing, China

**Keywords:** cell cycle regulatory gene, *β*-cyclodextrin-hemin complex (*β*-CDH), hydrogen peroxide, lateral root formation, *Solanum lycopersicum*

## Abstract

Although previous results showed that *β*-cyclodextrin-hemin complex (*β*-CDH) could induce tomato lateral root (LR) formation, the corresponding downstream messengers are still not fully understood. In this report, similar to the inducing effects of exogenously applied hydrogen peroxide (H_2_O_2_), we discovered that *β*-CDH elicited *RBOH1* transcript upregulation, endogenous H_2_O_2_ accumulation, and thereafter tomato LR development. Above responses were sensitive to dimethylthiourea (DMTU) and ascorbic acid (AsA), two membrane-permeable scavengers of H_2_O_2_, showing that accumulation of H_2_O_2_ and LR formation were significantly blocked. The test with diphenyleneiodonium (DPI; the inhibitor of NADPH oxidase) revealed that H_2_O_2_ mainly produced by NADPH oxidase, might be involved in LR formation triggered by *β*-CDH. qPCR combined with pharmacological and anatomical analyses showed that *β*-CDH-modulated several marker genes responsible for LR formation, such as *CYCA3;1, CYCA2;1, CYCD3;1*, and *CDKA1* (four cell cycle regulatory genes), *ARF7* and *RSI-1* (two auxin signaling genes), *LAX3* (an auxin influx carrier), *IAA14* (encoding a member of the Aux/IAA protein family), *PIN3* and *PIN7* (two auxin efflux carriers), *isocitrate dehydrogenase [NADP], NADH-cytochrome b_5_ reductase 1*, and *L-ascorbate oxidase homolog* genes (two reactive oxygen species-associated genes and one LR formation-related gene), were causally related to above H_2_O_2_ signaling. Particularly, representative proteins related to H_2_O_2_ metabolism and lateral rooting, were specifically induced in *β*-CDH-treated tomato seedlings. Overall, the results clearly suggested a vital role of H_2_O_2_ in the *β*-CDH-induced tomato LR formation, and *β*-CDH-elicited H_2_O_2_-related target proteins responsible for LR formation might be, at least partially, regulated at transcriptional and translational levels.

## Introduction

It was well known that lateral root (LR) not only acts as a physical support, but also enables plants to absorb and transport water and nutrients (Casimiro et al., 2003; Benková and Bielach, 2010). Since LR is a very important agronomic trait, the corresponding chemical inducers and corresponding mechanism of its formation have been widely studied (Fukaki and Tasaka, 2009). *β*-cyclodextrin-hemin (*β*-CDH), which combines hemin with β-cyclodextrin (β-CD), a cyclic oligosaccharide of seven *α*-(1,4) linked glucose units (Bodine et al., 2004), is previously discovered to be a novel inducer of LR formation in tomato seedlings (Li et al., 2015). Compared with hemin, the solubility of *β*-CDH in aqueous solution and its efficiency in inducing LR formation were significantly improved. Since the involvement of nitric oxide (NO), heme oxygenase-1 (HO-1), and glutathione (Li et al., 2015; Zhu et al., 2016) in above *β*-CDH response was respectively illustrated, the intricate signaling web triggered by *β*-CDH in LR formation is an excellent model. Corresponding mechanism may thus reveal that, how a vital agronomic trait elicited by an exogenous chemical is controlled by a complex array of signaling mechanisms.

Beside its toxic effects, ample evidence revealed that hydrogen peroxide (H_2_O_2_) can act as an important signaling molecule participating a series of physiological processes (Neill et al., 2002), including plant development (Li et al., 2011; Bai et al., 2012; Zhao et al., 2012), responses against abiotic and biotic stress (Levine et al., 1994; Alvarez et al., 1998; Desikan et al., 2004, 2006; Zhou et al., 2012, 2014; Rejeb et al., 2015), and even programmed cell death (Wu et al., 2011). In the downstream of H_2_O_2_ signal, proteomics changes as well as post-translational modifications are suggested as the important processes. For example, it was reported that protein expression and protein carbonylation were regulated in H_2_O_2_ signal response (Tanou et al., 2009, 2012; Lounifi et al., 2013). Although the role of respiratory burst oxidase homologs (RBOH)-mediated H_2_O_2_ as a second messenger in root organogenesis has been extensively illustrated (Li et al., 2007, 2009; Cao et al., 2014; Ma et al., 2014; Orman-Ligeza et al., 2016), it was not known whether H_2_O_2_ could act as an intermediate in *β*-CDH-induced LR formation. Meanwhile, several important downstream signaling components, including NO and HO-1 (Bai et al., 2012; Lin et al., 2012; Cao et al., 2014; Fang et al., 2014; Ma et al., 2014; Li et al., 2015), are shared in some aspects of *β*-CDH- and H_2_O_2_-induced root organogenesis. These results suggested the possibility that a linear pathway from *β*-CDH to H_2_O_2_ may exist in LR formation.

In this study, it was found that *β*-CDH elicited *RBOH1* transcript upregulation, endogenous H_2_O_2_ accumulation, and thereafter tomato LR development, mimicking the responses of exogenously applied H_2_O_2_. By using pharmacological, anatomical, and molecular approaches, we further revealed that H_2_O_2_ operates downstream of *β*-CDH promoting LR development. Additionally, H_2_O_2_ metabolism related proteins or other target proteins responsible for LR formation might be regulated by *β*-CDH at transcriptional and translational levels. Combined with the inducing responses in adventitious root development elicited by *β*-CDH (Lin et al., 2012), our results thus provided a comprehensive window of the complex signaling transduction pathway in *β*-CDH-mediated root organogenesis.

## Materials and Methods

### Chemicals

Unless stated otherwise, all chemicals were purchased from Sigma (St Louis, MO, United States). According to previous reports (Li et al., 2015; Zhu et al., 2016), the preparation of *β*-CD-hemin (*β*-CDH) was carried out. Hemin (used as an inducer of HO-1) and *β*-CD with an appropriate molar ratio were mixed by grinding for at least 60 min after adding de-ionized water. After freeze-dried, the brown powder was regarded as *β*-CDH. Our pilot experiment confirmed that 1 nM *β*-CDH which contains 1 nM hemin and 500 nM *β*-CD, exhibited a maximal response in the induction of tomato LR (Li et al., 2015).

Hydrogen peroxide (H_2_O_2_), as a positive control, was applied at 100 μM. *N,N′*-dimethylthiourea (DMTU; Ma et al., 2014), a membrane-permeable scavenger of H_2_O_2_, was used at a final concentration of 500 μM. Ascorbic acid (AsA; another membrane-permeable scavenger of H_2_O_2_) purchased from Solarbio Life Sciences (Beijing, China), was used at 200 μM. Diphenyleneiodonium (DPI), a NADPH oxidase inhibitor (Xie et al., 2011), was used at 0.1 μM. According to our pilot experiments, the concentrations of above chemicals exhibiting the effective responses were selected.

### Plant Material and Growth Conditions

Tomato (*Solanum lycopersicum* L.) seeds “baiguoqiangfeng” were obtained from Jiangsu Academy of Agricultural Sciences. Selected seeds were surface-sterilized with 2% NaClO for at least 10 min, and germinated in distilled water at 25 ± 1°C in the dark for 2 days. Afterward, tomato seedlings were transferred to an illuminating incubator (25 ± 1°C) with a light intensity of 200 μmol m^-2^ s^-1^ at 14-h photoperiod. After growing for 1 day, the selected identical seedlings were transferred to 4 ml solution containing the indicated chemicals for the indicated time points. Afterward, photographs were taken, and the number of emerged LRs (LRs; >1 mm) per seedling and the length of primary root (PR), as well as the emerged LR density (the number of LR per cm PR; LRs/cm) were determined with Image J software. LR primordia (LRP) per seedling were also observed by root squash preparations and quantified by a light microscope (model Stemi 2000-C; Carl Zeiss, Germany; Correa-Aragunde et al., 2006). In our test, at least three independent experiments were carried out for each treatment, and at least 15 seedlings were used for each.

For the subsequent biochemical, molecular and proteomics analyses, only the LR-inducible segments were used. Therefore, the root apical meristems of seedlings at the indicated time points were cut off, and the shoots were removed by cutting below the root-shoot junction (Zhu et al., 2016).

### H_2_O_2_ Detection and Fluorescence Analysis

H_2_O_2_ signals were assessed by a laser confocal scanning microscopy (LCSM) using the ROS fluorescent probe 2′,7′-dichlorofluorescein diacetate (H_2_DCF-DA) (Maffei et al., 2006; Li et al., 2011; Xie et al., 2011). Also, DMTU and AsA, two membrane-permeable scavengers of H_2_O_2_, were used to confirm its specificity. Roots were incubated in HEPES buffer (20 mM, pH 7.5) which contains 20 μM H_2_DCF-DA for 30 min in dark (25°C). Then the fresh HEPES buffer was used to wash three times. All images were visualized by using UltraVIEW VoX (Perkin Elmer, Waltham, MA, United States). Thereafter, photographs were representative of identical results obtained after the processing and analysis of seven samples for each condition in three independent experiments. Volocity Demo software was used to quantify the production of H_2_O_2_ in roots.

### Real-time Quantitative RT-PCR (qPCR) Analysis

Total RNA was isolated using the Trizol reagent (Invitrogen, Gaithersburg, MD, United States) according to the manufacturer’s instructions. The RNA samples were treated with RNAase-free DNase (TaKaRa Bio Inc., Dalian, China) to eliminate traces of DNA, followed by the quantification by using the NanoDrop 2000 (Thermo Fisher Scientific, Wilmington, DE, United States). Afterward, total RNA (2 μg) was reverse-transcribed using an oligo(dT) primer and M-MLV reverse transcriptase (BioTeke, Beijing, China).

Real-time qPCR reactions were performed using a Mastercycler^®^ ep *realplex* real-time PCR system (Eppendorf, Hamburg, Germany) with SYBR^®^*Premix Ex Taq*^TM^ (TransGen Biotech, Beijing, China) according to the manufacturer’s instructions. The primer sequence information was listed in Supplementary Table S1. Relative expression levels of corresponding genes were presented as values relative to the control samples at the indicated time points, after normalization with *Actin* transcript levels (Zhu et al., 2016).

### Proteomics Analysis

The total proteins in tomato root tissues were extracted by Plant Total Protein Extraction Kit (Sigma-Aldrich, St Louis, MO, United States). Protein samples (200 μg BSA equivalent) were digested using filter-aided sample preparation (FASP) method (Wiśniewski et al., 2009). The protein extraction was reduced with 10 mM dithiothreitol (DTT) for 1 h at 56°C, and then alkylated with 55 mM iodoacetamide (IAA) for 45 min at 25°C in darkness. Afterward, the protein samples were buffer-exchanged with 100 mM NH_4_HCO_3_ (pH 8.0–8.5) using 10 kDa molecular weight cut-off Amicon Spin Tube (Millipore, Billerica, MA, United States). Subsequently, 4 μg of sequencing-grade modified trypsin (Promega) was added to each sample, and digestion was carried out overnight at 37°C (trypsin: protein ration = 1: 50). Digested peptides were desalted by Ziptip C18 (Milipore) and quantified using a NanoDrop 2000 spectrophotometer (Wilmington, United States).

For LC-MS/MS conditions, a label-free quantitative method was used to detect the relative amount of proteins. Three biological replicates from three independent experiments (about 60 roots for each independent experiment) of *β*-CDH-treated and control groups were analyzed by nano LC system (Dionex, part of Thermo Fisher Scientific) on-line coupled to LTQ-Orbitrap mass spectrometer (Thermo Electron, Bremen, Germany). The resulting peptides (1.5 μg) were acidified with 0.1% formic acid (FA), and subsequently loaded onto the nano trap column (Acclaim PepMap100 C18, 75 μm × 2 cm, 3 μm, 100 Å, Thermo Scientific) at a flow rate of 4 μL⋅min^-1^ in loading buffer (2% acetonitrile, 0.1% FA in HPLC-grade water). Chromatographic separation was carried out on the analytical column (Acclaim PepMap^®^RSLC, C18, 75 μm × 15 cm, 3 μm, 100Å, Thermo Scientific) using a linear gradient of 3–55% buffer B (80% acetonitrile and 0.1% FA) at a flow rate of 0.25 μL⋅min^-1^ over 112 min. Due to loading and washing steps, the total time for an LC-MS/MS run was 160 min longer. For LTQ-Orbitrap analysis, one scan cycle included an MS1 scan (m/z 300–1800) at a resolution of 60,000, followed by 10 MS2 scans by LTQ, to fragment the 10 most abundant precursor ions at normalized collision energy of 35 eV. The lock mass calibration was activated, and dynamic exclusion time was set to 30 s.

Raw data were analyzed by MaxQuant (version 1.5.2.5) (Tyanova et al., 2016) using standard settings with the additional options match between runs, and LFQ selected. The generated ‘proteingroups.txt’ table was filtered for contaminants, reverse hits, and number of unique peptides (>0) in Perseus (from MaxQuant package).

### Data Analysis

Where indicated, results were expressed as the mean values ± SE of at least three independent experiments (with at least three replicates for each) with similar results. Statistical analysis was performed using SPSS 17.0 software. For statistical analysis, one-way analysis of variance (ANOVA) followed by Duncan’s multiple range test (*P* < 0.05) was chosen.

## Results

### Endogenous H_2_O_2_ Production Is induced by *β*-CDH

**FIGURE 1:**
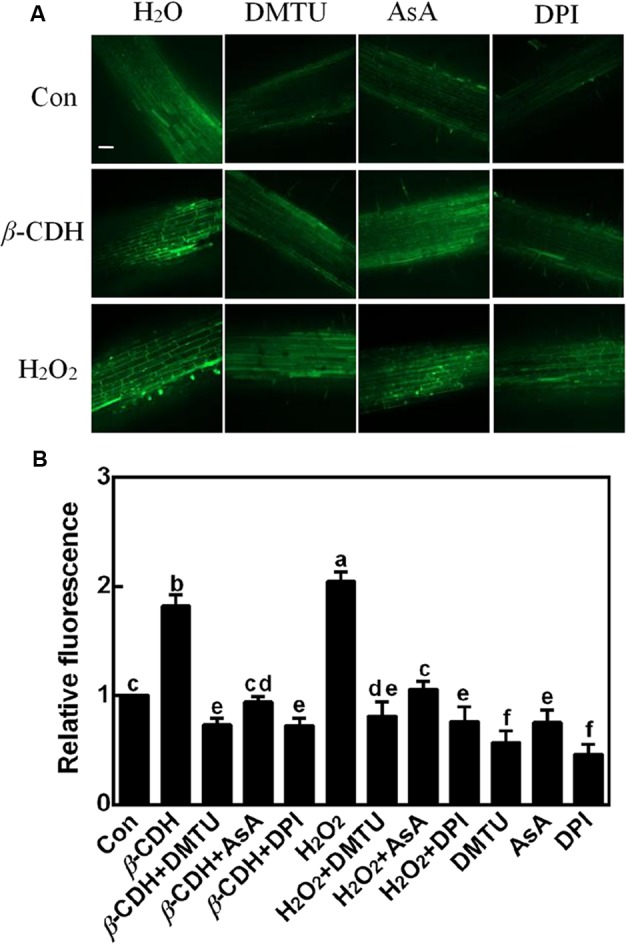
Changes of endogenous H_2_O_2_ production. Three-day-old tomato seedlings were treated with H_2_O (Con), 1 nM *β*-CDH, 100 μM H_2_O_2_, 500 μM DMTU, 200 μM AsA, and 0.1 μM DPI, alone or their combinations for 12 h. Corresponding confocal images of DCF-dependent fluorescence in seedling roots was shown in **(A)**. Bar = 55 μm. Meanwhile, the relative fluorescence **(B)** was presented as values relative to control. Mean and SE values were calculated from at least three independent experiments with at least three replicates for each. Bars denoted by the same letter did not differ significantly at *P* < 0.05 according to Duncan’s multiple range test.

First, tomato seedlings were loaded with reactive oxygen species (ROS)-specific fluorescent dye 2′,7′-dichlorofluorescein diacetate (H_2_DCF-DA), and LCSM was used to investigate changes in ROS-induced fluorescence. Since the DCF-dependent green fluorescence detected in 100 μM H_2_O_2_-treated tomato seedlings for 12 h, was obviously impaired following the addition of DMTU and AsA, two membrane-permeable scavengers of H_2_O_2_ (**Figure 1**), the visual signal can be mostly ascribed to endogenous H_2_O_2_ accumulation. Thus, the fluorescence was used to report endogenous H_2_O_2_ levels subsequently.

Further result showed that, compared to the control sample, the addition of 1 nM *β*-CDH for 12 h was able to induce endogenous H_2_O_2_ production in tomato seedlings, mimicking the response of H_2_O_2_ when was exogenously applied. We also noticed that this time point of H_2_O_2_ production triggered by *β*-CDH and exogenous H_2_O_2_, apparently preceded LR formation, beginning at 48 h of treatments (Li et al., 2015). Above results indicated the possible link between endogenous H_2_O_2_ production and LR formation triggered by *β*-CDH.

### The Removal of H_2_O_2_ Prevents *β*-CDH-Induced H_2_O_2_ Production and Thereafter LR Formation

**FIGURE 2:**
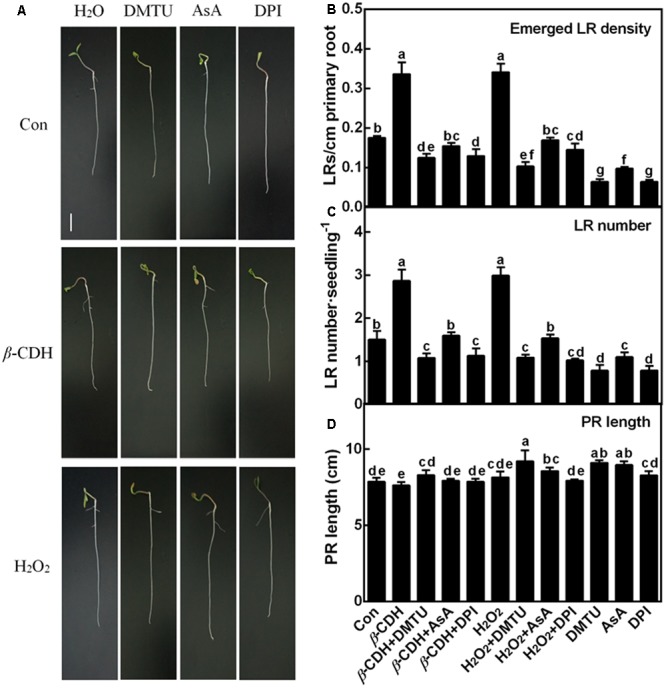
The lateral root formation triggered by *β*-CDH was blocked by H_2_O_2_ depletion. Three-day-old tomato seedlings were treated with H_2_O (Con), 1 nM *β*-CDH, 100 μM H_2_O_2_, 500 μM DMTU, 200 μM AsA, and 0.1 μM DPI, alone or their combinations for 4 days. Afterward, corresponding photographs were taken **(A)**. Bar = 1 cm. Meanwhile, the emerged LR density **(B)**, the number of emerged LRs (>1 mm) per seedling **(C)**, and the primary root (PR) length **(D)** were determined. Mean and SE values were calculated from at least three independent experiments with at least three replicates for each. Bars denoted by the same letter did not differ significantly at *P* < 0.05 according to Duncan’s multiple range test.

In order to evaluate the possible role of endogenous H_2_O_2_ in *β*-CDH-induced LR development, DMTU and AsA were also used. Similar to the previous reports (Ma et al., 2014; Li et al., 2015; Zhu et al., 2016), both 1 nM *β*-CDH and 100 μM H_2_O_2_ increased tomato LR density and number (**Figures 2A–C**). Meanwhile, no significant difference in PR length was observed (**Figure 2D**). By contrast, the co-treatment with DMTU and AsA respectively not only blocked endogenous H_2_O_2_ production (**Figure 1**), but also arrested the thereafter induction of LR formation (**Figure 2**), triggered by exogenous *β*-CDH and H_2_O_2_. When applied alone, DMTU (in particularly) and AsA could inhibit LR formation respect to the chemical-free control plants. Meanwhile, endogenous H_2_O_2_ levels were also decreased (**Figure 1**).

### Generation of H_2_O_2_ and Induction of Lateral Rooting by *β*-CDH Are Mediated Partly by NADPH Oxidase

For the origin of endogenous H_2_O_2_, the plasma-membrane (PM) NADPH oxidase confers important roles in H_2_O_2_ signaling (Desikan et al., 2006; Xie et al., 2011). Since DPI is an inhibitor of NADPH oxidase responsible for endogenous H_2_O_2_ production during LR formation (Ma et al., 2014), this chemical was applied together with *β*-CDH. Similar to the inhibition responses of DMTU and AsA (**Figures 1, 2**), *β*-CDH-induced H_2_O_2_ and LR formation were respectively impaired by 0.1 μM DPI, suggesting the possible role of NADPH oxidase-dependent H_2_O_2_ in *β*-CDH action. When applied alone, DPI, similar to DMTU (in particular) and AsA, could inhibit LR formation, compared to the chemical-free control plants (**Figure 2**). Changes in endogenous H_2_O_2_ displayed the similar tendencies (**Figure 1**).

### *β*-CDH- and H_2_O_2_-Triggered Lateral Root Primordial (LRP) Are Impaired by DMTU, DPI, and AsA

**FIGURE 3:**
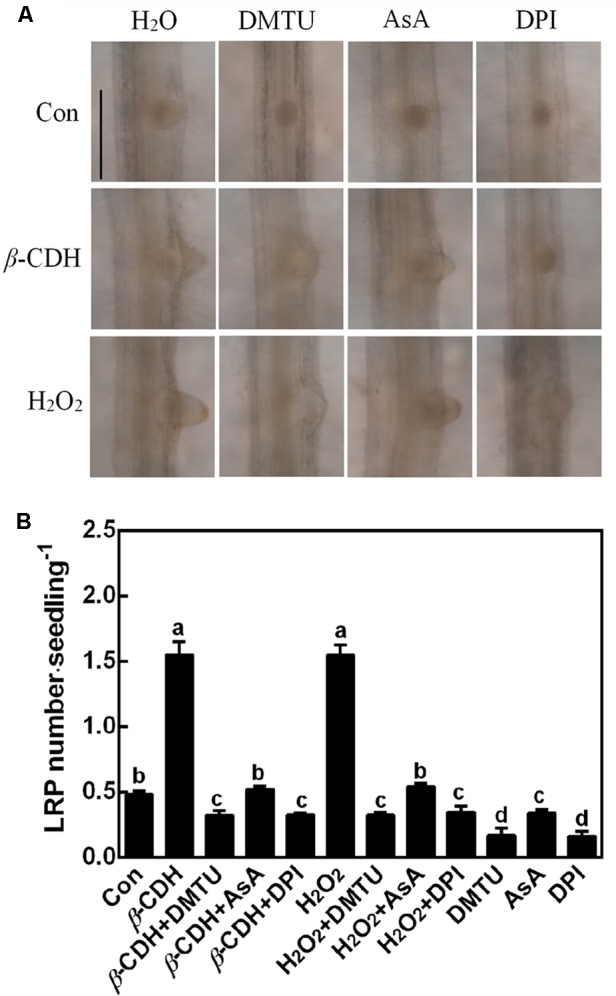
*β*-CDH-induced lateral root primordial (LRP) is sensitive to H_2_O_2_ depletion. Three-day-old tomato seedlings were treated with H_2_O (Con), 1 nM *β*-CDH, 100 μM H_2_O_2_, 500 μM DMTU, 200 μM AsA, and 0.1 μM DPI, alone or their combinations for 3 days. Afterward, photographs showing the representative morphology of LRP (about 75% of LRP at the shown stages) were taken **(A)**. Bar = 0.25 mm. Meanwhile, the number of emerged LRP was also analyzed **(B)**. Mean and *SE* values were calculated from at least three independent experiments with at least three replicates for each. Bars denoted by the same letter did not differ significantly at *P* < 0.05 according to Duncan’s multiple range test.

Further microscopical analysis showed that both *β*-CDH- and H_2_O_2_-triggered LR primordial (LRP; 3 days) exhibited a similar accelerated anatomic structure, both of which were individually impaired by the cotreatment with DMTU, DPI or AsA (**Figure 3**). When applied alone, DMTU, DPI, or AsA strongly inhibited the development of LRP. We also noticed that above results were comparable to the phenotypes in the LR formation (**Figure 2**).

### Both *β*-CDH- and H_2_O_2_-Up-Regulated *RBOH1* Are Sensitive to the Removal of H_2_O_2_

**FIGURE 4:**
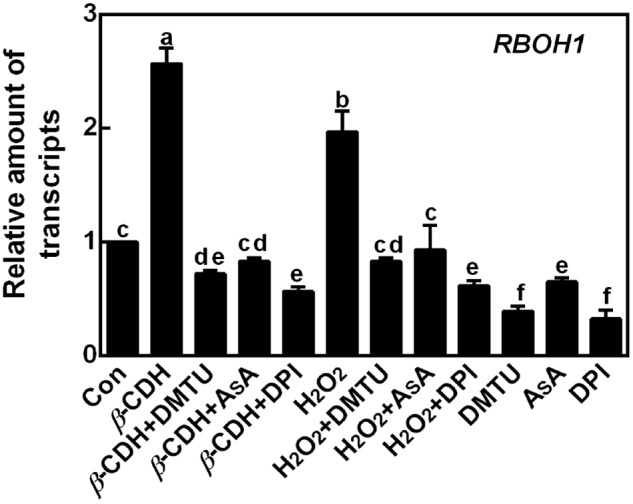
*β*-CDH-induced *RBOH1* transcript is blocked by H_2_O_2_ depletion. Three-day-old tomato seedlings were treated with H_2_O (Con), 1 nM *β*-CDH, 100 μM H_2_O_2_, 500 μM DMTU, 200 μM AsA, and 0.1 μM DPI, alone or their combinations for 6 h. Afterward, the amounts of *RBOH1* transcript were analyzed by qPCR, and presented relative to the control sample. Mean and SE values were calculated from at least three independent experiments with at least three replicates for each. Bars denoted by the same letter did not differ significantly at *P* < 0.05 according to Duncan’s multiple range test.

The inhibiting effect of DPI on *β*-CDH-elicited LR formation suggested the possible role of NADPH oxidase. The following experiments were carried out to test above hypothesis. As shown in **Figure 4**, the transcript of *RBOH1* was rapidly increased after *β*-CDH or exogenous H_2_O_2_ treatments for 6 h. Meanwhile, the removal of H_2_O_2_ (**Figure 1**) by the scavengers of H_2_O_2_ (DMTU and AsA) and inhibitor of NADPH oxidase (DPI) completely blocked above responses. Similarly, DMTU, AsA and DPI alone also exhibited the inhibition in *RBOH1* expression compared to control samples.

### The Transcripts of Target Genes Are Regulated by *β*-CDH and H_2_O_2_

**FIGURE 5:**
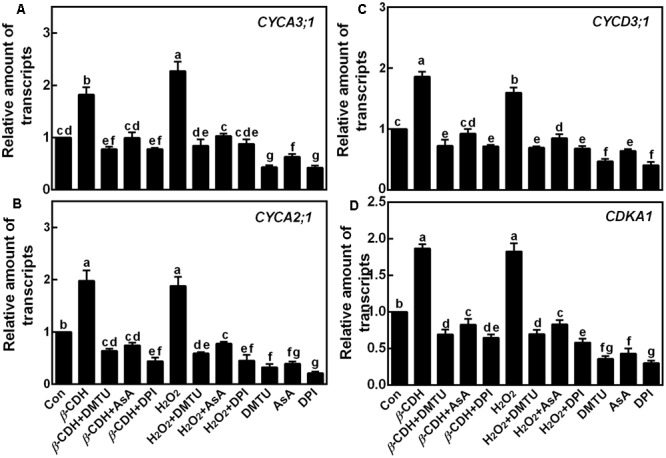
Changes of the transcripts of cell cycle genes. Three-day-old tomato seedlings were treated with H_2_O (Con), 1 nM *β*-CDH, 100 μM H_2_O_2_, 500 μM DMTU, 200 μM AsA, and 0.1 μM DPI, alone or their combinations for 12 h. Afterward, the amounts of *CYCA3;1* **(A)**, *CYCA2;1* **(B)**, *CYCD3;1* **(C)**, and *CDKA1* **(D)** transcripts were analyzed by qPCR, and presented relative to the control sample. Mean and SE values were calculated from at least three independent experiments with at least three replicates for each. Bars denoted by the same letter did not differ significantly at *P* < 0.05 according to Duncan’s multiple range test.

Furthermore, the transcripts of four cell cycle regulatory genes, *CYCA3;1, CYCA2;1, CYCD3;1*, and *CDKA1*, were analyzed by qPCR as molecular probes to further investigate the role of H_2_O_2_ in *β*-CDH-induced LR formation. After 12 h of *β*-CDH treatment, above transcripts were up-regulated (**Figure 5**). Similar results appeared in H_2_O_2_-treated seedlings. However, the addition with DMTU, AsA, or DPI could significantly prevent *β*-CDH- and H_2_O_2_-induced cell cycle regulatory gene expression, all of which were well matched with the LPR number, LR number and density (**Figures 2, 3**).

**FIGURE 6:**
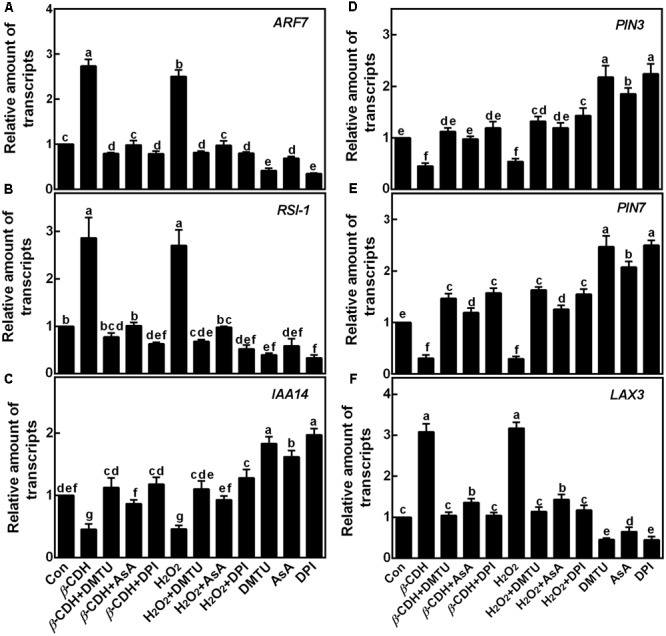
Changes of the transcripts of the target genes. Three-day-old tomato seedlings were treated with H_2_O (Con), 1 nM *β*-CDH, 100 μM H_2_O_2_, 500 μM DMTU, 200 μM AsA, and 0.1 μM DPI, alone or their combinations for 12 h. Afterward, the amounts of *ARF7* **(A)** and *RSI-1* (**B**; two auxin signaling genes), *IAA14* (**C**; encoding a member of the Aux/IAA protein family), *PIN3* **(D)** and *PIN7* (**E**; two auxin efflux carriers), and *LAX3* (**F**; an auxin influx carrier) transcripts were analyzed by qPCR, and presented relative to the control sample. Mean and SE values were calculated from at least three independent experiments with at least three replicates for each. Bars denoted by the same letter did not differ significantly at *P* < 0.05 according to Duncan’s multiple range test.

Subsequent experiment revealed that *β*-CDH and H_2_O_2_ were able to up-regulate the transcripts of auxin signaling genes (*ARF1* and *RSI-1*), and an auxin influx carrier gene (*LAX3*), together with the down-regulation of *IAA14* (encoding a member of the Aux/IAA protein family) and two auxin efflux carriers genes (*PIN3* and *PIN7*; **Figure 6**). As we expected, the co-treatment with DMTU, AsA, or DPI differently blocked the above mentioned effects. Combined with corresponding endogenous H_2_O_2_ production (**Figure 1**) and phenotypes (**Figures 2, 3**), these findings suggested that above genes might be the targets of H_2_O_2_ signaling in *β*-CDH-induced tomato LR formation.

### Comparative Proteomic Analysis and Its Transcription Confirmation

To well address molecular mechanism of *β*-CDH-induced LR formation, comparative proteomic analysis from tomato seedling roots in the presence or absence of *β*-CDH was performed with LC-MS/MS. In this study, a total of 86 proteins were identified significantly regulated (fold change > 1.5 or < 0.667) after *β*-CDH treatment under *P* value < 0.05 (Supplementary Table S2). Some ROS metabolism related proteins were modulated by *β*-CDH treatment, such as Isocitrate dehydrogenase [NADP] (decreased), Catalase, Succinic semialdehyde reductase isofom1 (SSR1), NADH-cytochrome *b_5_* reductase 1 (increased), etc (**Table 1**). Meanwhile, proteins related to LR formation, like L-ascorbate oxidase (increased), Protein ROOT HAIR DEFECTIVE 3, DWARF1/DIMINUTO, Phenylalanine ammonia-lyase, and Glycine rich RNA binding protein 1a, were found be regulated after *β*-CDH treatment. Furthermore, 20 proteins like Malic enzyme, Coatomer subunit alpha, PR10 protein, and 40S ribosomal protein S8, etc. were identified to be working in other biological process, such as metabolic process, intracellular transport, response to stress, and protein metabolic process, etc. Additionally, we also noticed that membrane-associated NADPH oxidase protein was not found in our experimental conditions.

**Table 1:** Proteins in tomato seedling roots that differentially expressed greater than 1.5-fold or less than 0.667-fold after *β*-CDH treatment for 48 h using MaxQuant analysis.

No.	Uniport accession no.	Unique peptides	Protein name	Ratio (*β*-CDH/Control)	*P*-value
**ROS metabolism related proteins**					
1	K4ASC2	9	Isocitrate dehydrogenase [NADP]	0.497297	0.000837
2	K4BAE6	10	Catalase	0.656807	0.03302
3	K4D331	5	NADH-cytochrome *b_5_* reductase 1	1.623331	0.039992
4	B1Q3F6	6	Succinic semialdehyde reductase isofom1 (SSR1)	0.65489	0.049261
5	K4C0T5	1	Peroxidase	0.53012	0.01681
6	Q9LWA2	2	Peroxidase	0.219145	0.027241
7	K4AZL9	4	Cysteine synthase	0.664404	0.006588
**LR formation related proteins**					
8	K4DH18	7	L-ascorbate oxidase homolog	2.284058	0.005003
9	K4BMV8	8	Protein ROOT HAIR DEFECTIVE 3 homolog	1.975167	0.001832
10	Q66YT8	11	DWARF1/DIMINUTO	1.57688	0.015569
11	K4D451	4	Phenylalanine ammonia-lyase	1.51914	0.026422
12	L7Q568	2	Glycine rich RNA binding protein 1a	0.390727	0.007481
**Other proteins**					
13	O04936	15	Malic enzyme	0.660573	0.005068
14	Q96480	6	Delta-1-pyrroline-5-carboxylate synthase	1.719958	0.026624
15	G8Z278	6	Hop-interacting protein THI111	2.223662	0.033841
16	K4BP97	5	Proteasome subunit beta type	0.656074	0.041904
17	O04870	8	Pectinesterase	1.690174	0.008905
18	K4DCH1	4	Ketol-acid reductoisomerase	0.574139	0.007607
19	O82575	2	Fruit-ripening protein	0.25713	0.001204
20	K4BVH7	7	Coatomer subunit alpha	1.677745	0.037297
21	V5YN09	5	Plasma membrane intrinsic protein 26	0.398124	0.019138
22	K4CWC5	4	PR10 protein	0.615877	0.016646
23	P12670	2	Protein NP24	1.513811	0.001122
24	P27065	4	Ribulose bisphosphate carboxylase large chain	0.577539	0.013686
25	P35057	4	Histone H4	0.638498	0.013939
26	K4C793	2	Ribosomal protein L15	0.60878	0.009944
27	K4CAH3	4	40S ribosomal protein S8	0.561525	0.01883
28	P49215	7	40S ribosomal protein S17	1.967542	0.01852
29	K4AWT4	2	40S ribosomal protein S21	0.343903	0.00246
30	K4BU29	1	40S ribosomal protein S21	0.327242	0.03347
31	P46301	2	40S ribosomal protein S25	0.383874	0.028931
32	K4AT06	2	40S ribosomal protein S27	1.683614	0.008617

**FIGURE 7:**
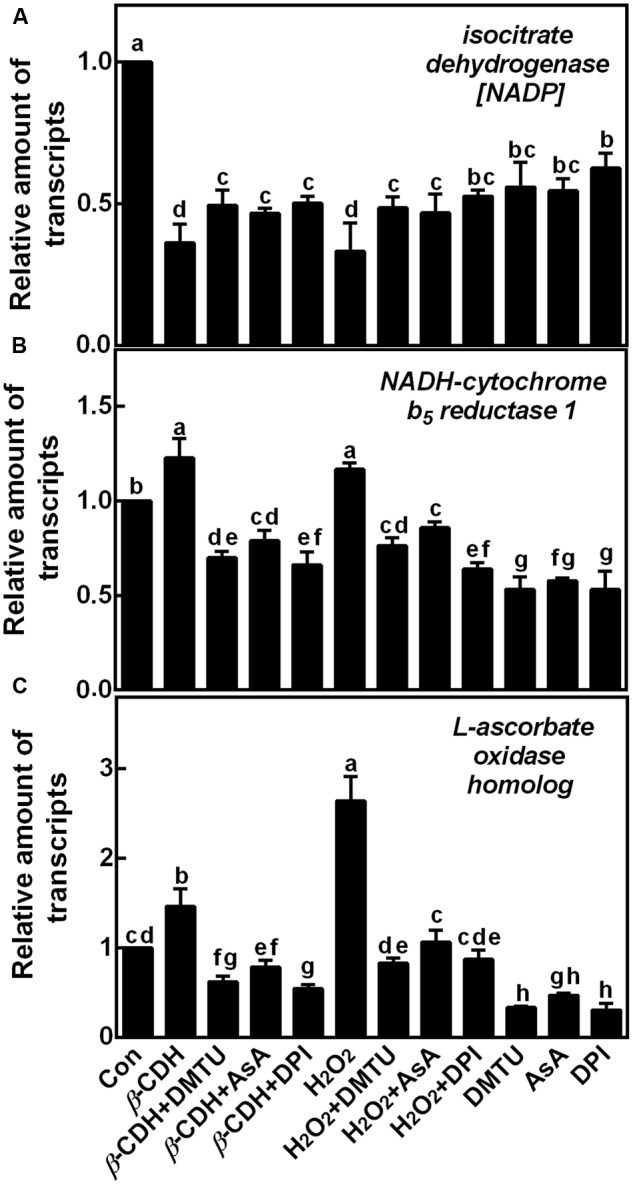
Changes of corresponding gene transcripts related to ROS metabolism and LR formation. Three-day-old tomato seedlings were treated with H_2_O (Con), 1 nM *β*-CDH, 100 μM H_2_O_2_, 500 μM DMTU, 200 μM AsA, and 0.1 μM DPI, alone or their combinations for 36 h. The relative amount of *isocitrate dehydrogenase [NADP]* **(A)**, *NADH-cytochrome b_5_ reductase 1* **(B)**, and *L-ascorbate oxidase homolog* **(C)** transcripts were detected by qPCR, and presented relative to the control sample. Mean and SE values were calculated from three independent experiments with at least three replicates for each. Bars denoted by the same letter did not differ significantly at *P* < 0.05 according to Duncan’s multiple test.

To confirm above results, we further tested the effects of H_2_O_2_ scavengers and inhibitor on the transcripts of three representative genes (**Table 1**), *isocitrate dehydrogenase [NADP], NADH-cytochrome b_5_ reductase*, and *L-ascorbate oxidase homolog* (**Figure 7**). Results showed that, the added H_2_O_2_ scavengers (DMTU and AsA) and synthetic inhibitor (DPI) could effectively prevent the down-regulation of *isocitrate dehydrogenase [NADP]* gene expression elicited by *β*-CDH and H_2_O_2_. Whereas, the up-regulated *NADH-cytochrome b_5_ reductase* and *L-ascorbate oxidase homolog* (in particular) transcripts were blocked. These results could be well consistent with the data form LC-MS/MS (**Table 1**).

## Discussion

Although the induction of LR formation by *β*-CDH was reported in previous studies (Li et al., 2015; Zhu et al., 2016), the detailed molecular mechanism is still not fully elucidated. In this report, we further show that endogenous H_2_O_2_ is involved in *β*-CDH-mediated LR formation in tomato seedlings, and *β*-CDH-elicited H_2_O_2_-related target proteins responsible for LR formation might be, at least partially, regulated at transcriptional and translational levels.

### H_2_O_2_ Is Involved in *β*-CDH-Induced LR Formation

It was well-known that H_2_O_2_ functions as a signaling molecule in regulating stress responses, development, and other cell processes (Neill et al., 2002; Cuypers et al., 2016; Niu and Liao, 2016). As expected, in our experimental condition, an increased endogenous H_2_O_2_ was induced by *β*-CDH in tomato seedlings (**Figure 1**). Evidences showed that, there are several enzymes that can produce H_2_O_2_ in plants, such as cell wall peroxidase, amine oxidase, flavin-containing enzymes, and NADPH oxidase in particularly (Cona et al., 2006; Xie et al., 2011; Francoz et al., 2015; Niu and Liao, 2016). We further identified that *β*-CDH-elicited H_2_O_2_ production resulted from the up-regulation of *RBOH1* gene expression (**Figure 4**). The involvement of NADPH oxidase in *β*-CDH-triggered LR formation was further corroborated by the findings that NADPH oxidase inhibitor DPI (Bai et al., 2012; Ma et al., 2014) not only inhibited H_2_O_2_ production (**Figure 1**), but also caused a significant reduction of LR formation in *β*-CDH-treated seedlings (**Figures 2, 3**). Meanwhile, the removal of endogenous H_2_O_2_ by DMTU and AsA exhibited the similar blocking tendencies, further confirming that LR formation elicited by *β*-CDH is closely related to endogenous H_2_O_2_ concentration. Although we can not exclude the possibility that these chemical agents used in the present study may not specifically target H_2_O_2_, these results clearly revealed that *β*-CDH-stimulated H_2_O_2_-mediated LR formation was partly dependent on NADPH oxidase. Consistently, a recent genetic result revealed that *RBOH*-mediated ROS production facilitated LR emergence in Arabidopsis (Orman-Ligeza et al., 2016).

Cell cycle activation is an important event during LR formation (Himanen et al., 2002, 2004; Casimiro et al., 2003). In the pervious reports, cell cycle genes *CYCA3;1, CYCA2;1, CYCD3;1*, and *CDKA1* were used as the molecular markers in tomato LR formation (Correa-Aragunde et al., 2006; Xu et al., 2011). Similar to the previous results (Li et al., 2015; Zhu et al., 2016), *β*-CDH treatment up-regulated *CYCA3;1, CYCA2;1, CYCD3;1*, and *CDKA1* gene expression, and these responses mimicked the effects of H_2_O_2_ (**Figure 5**; Ma et al., 2014). By contrast, above *β*-CDH- and H_2_O_2_-induced expression of cell cycle regulatory genes were prevented or delayed when H_2_O_2_ was scavenged by DMTU or AsA, or its synthesis was inhibited with DPI (Figure 1). Combined with the impaired LRP and LR formation by the removal of H_2_O_2_ levels (**Figures 1–3**), our findings gave further evidence, suggesting that *β*-CDH-triggered H_2_O_2_ production was able to modulate the expression of cell cycle regulatory genes and this event is also required for LR formation in tomato seedlings.

Auxin controls cell cycle progression and asymmetric divisions during LR formation (Casimiro et al., 2001; Bhalerao et al., 2002; Lavenus et al., 2013). In our experimental conditions, it was further confirmed that, similar to the responses elicited by H_2_O_2_, *β*-CDH up-regulated two auxin signaling genes (*ARF7* and *RSI-1*; Zhu et al., 2016) and an auxin influx carrier gene (*LAX3*), together with the down-regulation of *IAA14* (encoding a member of the Aux/IAA protein family) and two auxin efflux carriers genes (*PIN3* and *PIN7*; **Figure 6**). Comparatively, the removal of endogenous H_2_O_2_ drastically impaired corresponding changes conferred by *β*-CDH and H_2_O_2_. Previous results showed that *slr-1*, a gain-of-function mutant of *IAA14* exhibited a crucial defect in LR formation in Arabidopsis (Fukaki et al., 2002). Three *Arabidopsis thaliana* mutants, *lax3, pin3* and *pin7*, which are defective in auxin influx and efflux proteins, showed reduced or increased LR formation (Swarup et al., 2008; Lewis et al., 2011). Combined with previous genetic results, our molecular and pharmacologic evidence further indicated a possible link between *β*-CDH-induced H_2_O_2_-mediated LR formation and auxin signaling. This deduction should be investigated at genetic levels in the near future.

### Proteomic Analysis Revealed the Target Proteins in the Process of *β*-CDH-Stimulated LR Formation

Proteomic analysis showed the presence of 86 proteins which were significantly regulated by *β*-CDH treatment for 48 h (Supplementary Table S2). Among these proteins, some were concerned with ROS signaling (**Table 1**). For example, Isocitrate dehydrogenase [NADP] (NADP-ICDH; EC 1.1.1.42; K4ASC2) catalyzes oxidative decarboxylation of isocitrate to 2-oxoglutarate using NADP^+^ to form NADPH, and the latter is an important cofactor in many biosynthesis pathways and important in cellular defense against oxidative damage (Lee et al., 2002). It was reported that, NADP-ICDH can be damaged by ROS, and the inactivation of ICDH may lead to the perturbation of the antioxidant defense system in many cell process (Lee S.M. et al., 2001). In our experimental conditions, the amount of NADP-ICDH protein was decreased after *β*-CDH treatment (**Table 1**), which was in line with the increased ROS in seedling roots (**Figure 1**). Similarly, the protein level of the major H_2_O_2_ scavenging enzyme, catalase (CAT; EC 1.11.1.6; K4BAE6), was also decreased by *β*-CDH. This was consistent with a higher concentration of H_2_O_2_ in tomato seedling roots after *β*-CDH treatment, because the altering of CAT level can modulate H_2_O_2_ levels in plant cells (Vandenabeele et al., 2004). NADH-cytochrome *b_5_* reductase (K4D331) is found to play a key role in the NADH-dependent reduction of D-erythroascorbyl free radical, and can be active in the oxidative stress response of *Saccharomyces cerevisiae* (Lee J.S. et al., 2001). In HeLa cells, H_2_O_2_ -regulated expression of NADH-cytochrome *b*_5_ reductase was previously reported (Bello et al., 2003). In this study, the level of NADH-cytochrome *b_5_* reductase protein was increased when *β*-CDH was supplied, also confirming that a rapid H_2_O_2_ production appeared in seedling roots (**Figure 1**).

Besides the changes of H_2_O_2_ and redox related proteins, the *β*-CDH could regulate some proteins related to LR formation, for example, _L_-ascorbate oxidase (AO; EC 1.10.3.3; K4DH18), Protein ROOT HAIR DEFECTIVE 3 homolog (RHD3; K4BMV8), DWARF1/DIMINUTO (Q66YT8), Phenylalanine ammonia-lyase (PAL; EC 4.3.1.5; K4D451), and Glycine rich RNA binding protein 1a (atRZ-1a; L7Q568). The activity and expression of AO are closely correlated with cell expansion, which implies a role in cell wall loosening, cell division, and cell elongation (Kato and Esaka, 1999; Sanmartin et al., 2003). In this study, the level of AO protein was induced by *β*-CDH (**Table 1**), and there might be a link between AO expression and *β*-CDH-induced lateral rooting. Protein ROOT HAIR DEFECTIVE 3 (RHD3; K4BMV8) encodes an 89 kD polypeptide with putative GTP-binding motifs, with a common function in eukaryotic cell enlargement (Wang et al., 1997). The *rhd3* mutation alters the size of roots and root hairs. Here, our results showed that RHD3 homolog protein level was increased by *β*-CDH. *DWARF1/DIMINUTO* (Q66YT8) gene encodes a protein involved in steroid as well as brassinosteroid (BR) synthesis (Klahre et al., 1998). BRs are known interacting with auxin to promote LR development in Arabidopsis (Bao et al., 2004). Thus, the elevated DWARF1/DIMINUTO protein level can help to lateral rooting. Phenylalanine ammonia-lyase (PAL; EC 4.3.1.5; K4D451) is reported to be highly regulated during development and xylogenesis with the cell wall polymer lignin (Elkind et al., 1990). An increased PAL protein level was found during *β*-CDH-induced lateral rooting (**Table 1**). Glycine rich RNA binding protein 1a (atRZ-1a; L7Q568) over-expression plants showed delayed germination and seedling growth under salt and drought stresses (Kim et al., 2007). Since abiotic stress could induce LR formation, an important phenomenon of the stress-induced morphogenic response (SIMR) in plants (Potters et al., 2007), we further deduced that the decreased level in atRZ-1a protein by *β*-CDH might lead to a positive influence in LR formation. Additionally, changes in three representative genes related to ROS metabolism and LR formation, including *isocitrate dehydrogenase [NADP]* (A), *NADH-cytochrome b_5_ reductase 1* (B), and *L-ascorbate oxidase homolog* (**Figure 7**), approximately matched with corresponding proteomic data.

In summary, our results showed a vital role of H_2_O_2_ in the *β*-CDH-induced tomato LR formation, and *β*-CDH-elicited H_2_O_2_-related target proteins might be, at least partially, regulated at transcriptional and translational levels.

## Author Contributions

Conception and design of the study: WC, DZ, and LH. Acquisition of data for the study: WC, DZ, WenS, YM, DH, YS, YR, WeiS, QG, and DX. Analysis of data for the work: WC, DZ, and LH. Interpretation of data for the work: WC, DZ, WenS, WeiS, and LH. All authors read and approved the final manuscript.

## Conflict of Interest Statement

The authors declare that the research was conducted in the absence of any commercial or financial relationships that could be construed as a potential conflict of interest.
