# Supplementary material for: Hydrogen Peroxide Is Involved in β-Cyclodextrin-hemin Complex-Induced Lateral Root Formation in Tomato Seedlings
Source: Front Plant Sci. 2017 Aug 18;8:1445. doi: 10.3389/fpls.2017.01445 (PMC5563380; doi:10.3389/fpls.2017.01445)
Supplement: Supplementary file 1 [file Table_1.DOC]

**Table S1. The primer sequences of qPCR**

| Primer names | Accession number | Sequences (5’→3’) |
| --- | --- | --- |
| *Actin* | NM_001330119.1 | F: CCACGAGACTACATACAA |
|  |  | R: TACCACCACTGAGCACAA |
| *RBOH1* | NM_001247197.2 | F: AAGGAGTGGAGGGTGTGACT |
|  |  | R: AATGAGGCTCCGCCTAAACC |
| *CYCA2;1* | AJ243452 | F: CATTAACAAGGGTATGCGAA |
|  |  | R: GTCAGGTAAAGAGTGTCCGG |
| *CYCA3;1* | AJ243453 | F: TGCGGTTCTTGCCATCA |
|  |  | R: CGCCCAGTTGCTTCCA |
| *CYCD3;1* | AJ245415 | F: GGTCATTGCTTACTATGGCT |
|  |  | R: AAAAGGGGAACTTGGGTCTC |
| *CDKA1* | Y17225 | F: CACTTGCCTGTCGCCTCCTC |
|  |  | R: ACCCCCTCGTCTTCCTGCTC |
| *ARF7* | EF121545.1 | F: GGCGTAATCTTCAGGTAGG |
|  |  | R: ACTTGGCTGCTTAGGAAAC |
| *RSI-1* | NM_001247737.1 | F: TGTTCTTGATTTTGCTCAC |
|  |  | R: TTTGTTGCCATAAACTCCT |
| *IAA14* | NM_001279141.1 | F: GTGGTGGCGGAGGTGAATTA |
|  |  | R: GCAGGTGGCTTGATTGGATCT |
| *PIN3* | NM_001247248.1 | F: TACGCGATGATGGGGTTTCC |
|  |  | R: TTTGTTGTTGCTGGTGCTGG |
| *PIN7* | NM_001247275.1 | F: CTGCTATGCCTGCTGCTAGT |
|  |  | R: GGACCAAGCAAGACCAAGGA |
| *LAX3* | NM_001247753.1 | F: CTAGAGAGAACGCGGTGGAG |
|  |  | R: TGACCATACTTGCCCACCCT |
| *isocitrate dehydrogenase [NADP]* | XM_004228559.2 | F: GTGTCCTGATGGCAAGACCA |
|  |  | R: CCACGAGTCCAGGCAAAGAT |
| *NADH-cytochrome b_5_ reductase 1* | XM_010313891.1 | F: CCCAAGGGGCGCTTTAAGTA |
|  |  | R: TCGTCACGTCGTTGGGATTT |
| *_L_-ascorbate oxidase homolog* | XM_004253085.2 | F: AGGATCCTCAGTAGGCCTGG |
|  |  | R: AGCGCGATTAGGACCTTGAG |
